# Plasma Levels of Myonectin But Not Myostatin or Fibroblast-Derived Growth Factor 21 Are Associated with Insulin Resistance in Adult Humans without Diabetes Mellitus

**DOI:** 10.3389/fendo.2018.00005

**Published:** 2018-01-31

**Authors:** Freddy J. K. Toloza, Jose O. Mantilla-Rivas, Maria C. Pérez-Matos, Maria L. Ricardo-Silgado, Martha C. Morales-Alvarez, Jairo A. Pinzón-Cortés, Maritza Pérez-Mayorga, Martha L. Arévalo-Garcia, Giovanni Tolosa-González, Carlos O. Mendivil

**Affiliations:** ^1^Diabetes, Lipids and Metabolism Laboratory, School of Medicine, Universidad de los Andes, Bogotá, Colombia; ^2^Molecular Epidemiology of Endocrine Diseases Group, School of Medicine, Universidad Militar Nueva Granada, Bogotá, Colombia; ^3^Section of Endocrinology, Fundación Santa Fe de Bogotá, Bogotá, Colombia

**Keywords:** myokine, myostatin, myonectin, fibroblast-derived growth factor 21, insulin resistance, obesity, muscle, diabetes mellitus

## Abstract

**Background:**

Myokines are a group of protein mediators produced by skeletal muscle under stress or physical exertion. Even though their discovery and effects in cell culture and animal models of disease have elicited great enthusiasm, very little is known about their role in human metabolism. We assessed whether plasma concentrations of three known myokines [myonectin, myostatin, and fibroblast-derived growth factor 21 (FGF-21)] would be associated with direct and indirect indicators of insulin resistance (IR) in individuals who did not have a diagnosis of diabetes.

**Methods:**

We studied 81 adults of both sexes comprising a wide range of body adiposity and insulin sensitivity. All participants underwent a thorough clinical assessment and a 5-point oral glucose tolerance test with calculation of multiple IR and insulin sensitivity indices. Twenty-one of them additionally underwent a hyperinsulinemic–euglycemic clamp with determination of steady-state whole-body insulin-stimulated glucose disposal (“M”). We compared plasma myokine concentrations across quartiles of IR indices and clinical IR surrogates, and explored the correlation of each myokine with the *M*-value.

**Results:**

Plasma myonectin levels increased monotonically across quartiles of the incremental area under the insulin curve (higher values indicate more IR) (*p*-trend = 0.021) and decreased monotonically across quartiles of the insulin sensitivity index (ISI – higher values indicate less IR) (*p*-trend = 0.012). After multivariate adjustment for other relevant determinants of IR (body mass index, age, and sex), the negative association of myonectin with ISI persisted (standardized beta = −0.235, *p* = 0.023). Myostatin was not associated with any clinical IR indicator or direct IR index measure. In multivariate analyses, FGF-21 showed a trend toward a positive correlation with glucose disposal that did not reach statistical significance (standardized beta = 0.476, *p* = 0.091).

**Conclusion:**

The secretion of myonectin may constitute an attempt at a compensatory mechanism against IR in humans.

## Introduction

Insulin resistance (IR) is a key pathogenic mechanism for type 2 diabetes (DM2) and multiple other diseases. Recent evidence suggests that skeletal muscle is not only a target of insulin action but also a relevant endocrine organ as well. When muscle tissue is under stress it responds by secreting myokines, proteins with the ability to influence inflammation, glucose disposal, and adipose tissue phenotype *via* paracrine, autocrine, and possibly endocrine signaling (1). Despite their great potential as key metabolic players, our understanding of the role of myokines on human physiology and disease is still extremely limited. We aimed to study the association of circulating levels of three myokines: myostatin, myonectin, and fibrobast-derived growth factor 21 (FGF-21), with objective measures of IR in adult humans.

Myostatin was discovered in 1997 after a targeted search for proteins of the transforming growth factor superfamily (2). Myostatin is a negative regulator of the differentiation of myoblasts to myotubes *in vitro* (3), and myotubes from obese women secrete more myostatin than those of lean counterparts (4). Studies in murine models have found less inflammation and obesity-induced IR in myostatin knockout animals (5). Furthermore, antibodies against myostatin prevented age-induced sarcopenia in mice (6). However, the evidence about the influence of myostatin on insulin action and IR seems contradictory: Plasma myostatin decreases with increasing number of metabolic syndrome criteria in human patients (7). The relationship between myostatin and insulin action in humans is largely unknown.

Myonectin (also called CTRP15—C1q/TNF-related protein 15) was discovered in 2011 by Seldin et al. (8). Myonectin was shown to improve fatty acid (FA) uptake by cultured hepatocytes (8) and to increase in plasma of women after aerobic exercise training (9). Of note, during early stages of research, another protein (CTRP5) was also named myonectin. However, since CTRP15 is more selectively secreted by muscle tissue, the name myonectin was assigned to CTRP15 (8). Very little is known about the impact of myonectin on whole-body insulin action in humans.

Fibroblast growth factor 21 (FGF-21) is a pleiotropic hormone secreted mostly by the liver (10) but also by muscle under stress (11). FGF-21 activates brown adipose tissue thermogenesis in humans (12). FGF-21 levels correlate positively with body adiposity in humans (13), and are paradoxically higher in patients with the metabolic syndrome (14). Plasma FGF-21 levels increase in obese subjects and correlate positively with homeostasis model assessment of insulin resistance (HOMA-IR) (15). Additionally, when obese subjects experience weight loss with dietary or surgical intervention, FGF-21 levels decrease and IR-associated features improve (16). Preliminary human trials have shown positive effects of FGF-21 administration on metabolic parameters (17). These apparently contradicting findings make it relevant to understand the association of FGF-21 with directly measured IR in humans.

Given the potential but still unknown influence of myokines on nutrient and energy metabolism, we aimed to evaluate the association of circulating levels of myostatin, myonectin, and FGF-21 with whole-body IR in individuals without known diabetes mellitus. We also explored the association of these myokines with IR-associated features in our study participants.

## Materials and Methods

### Study Subjects

We studied 81 adults of both sexes, aged 30–69, residents of Bogotá, Colombia, who had no prior diagnosis of diabetes mellitus. Sampling was not probabilistic and was done by open convocation through various channels (email, posters, referred by other participants). However, we intentionally included patients with a wide range of body mass indexes (BMIs) and presumably different IR status. Besides known diabetes, other exclusion criteria were pregnancy, use of anti-diabetic medications, endocrine diseases, and use of anticoagulants or metformin for any indication. We also excluded patients who were acutely ill, or whose plasma high-sensitivity C-reactive protein (hsCRP) was above 10 mg/L. All subjects provided written informed consent.

We estimated the study’s power with the expression for cross-sectional studies that estimate a linear correlation coefficient (18). Given our study sample, the probability of finding a true linear correlation of at least 0.3 between the insulin sensitivity index by Gutt (ISI-Gutt) and each myokine under a 5% probability of type I error was 82% for myonectin, 84.5% for myostatin, and 83% for FGF-21.

The study was approved by the Internal Review Board (Comité de Ética) of Universidad de los Andes according to minute 307 of 2014. The study complied with scientific, technical and administrative norms for health research dictated by resolution 008430—1993 of the Colombian Ministry of Health and with the principles stated by the Declaration of Helsinki. All study subjects underwent an informed consent procedure and provided written informed consent.

### Clinical Assessment, Oral Glucose Tolerance Test (OGTT), and Hyperinsulinemic Clamp

We measured in all participants resting blood pressure, height, weight and abdominal circumference. Total and segmental percent body fat, percent lean mass, absolute lean mass (kg), and percent abdominal fat were determined with a tetrapolar biological impedance meter (BC545, TANITA^®^). This device estimates lean body mass (LBM) by measuring electrical impedance across two pairs of electrodes (hands and feet soles) and then inputting the measured impedance plus the gender, age and height into patented equations developed and validated in several thousand participants of different ethnicities, to predict percent LBM. Fasting blood samples were obtained in potassium oxalate tubes for measurement of fasting glucose and in EDTA tubes for all other determinations including myokines. After prompt plasma separation, a protease inhibitor cocktail was added and total plasma was separated in aliquots for each myokine and frozen at −80°C until analyzed.

For the OGTT patients arrived after 8–12 h overnight fast, and received a load of 75 g of glucose diluted in 300 mL of water to be consumed in less than 5 min. Blood samples were drawn for the measurement of plasma glucose and insulin measurement at times 0, 30, 60, 90, and 120 postglucose challenge. Patients could not smoke, ingest food or do significant physical activity during the OGTT. We selected completely at random a study subsample of 21 participants who additionally underwent a hyperinsulinemic–euglycemic clamp (19). After an overnight fast, subjects were admitted to a clinical research center, where two IV catheters were placed in the antecubital area of both arms for insulin and dextrose infusion. Another catheter was placed in the dorsal area of the hand ipsilateral to the dextrose infusion for blood sampling. The hand for capillary blood sampling and glucose measurement was placed inside a heated-hand box (The University of Vermont Medical, USA). This device allows the maintenance of a constant controlled temperature (56–58°C) in order to arterialize venous blood. A short-acting human insulin analog (Humalog, Lilly) was infused at an initial priming rate of 100 mU/m^2^/min that was reduced to 90 mU/m^2^/min after 2 min and then in 20 mU/m^2^/min steps every 2 min until a dose of 40 mU/m^2^/min was reached. This rate was then kept constant for the next 110 min, the whole procedure lasted 120 min. A variable infusion of 20% glucose was started at the fourth minute and adjusted every 5 min in order to maintain the arterialized venous glucose concentration at 100 mg/dL (95–105 mg/dL). Plasma glucose was measured by the glucose oxidase method using Accu-Chek Performa glucose meters (Roche, Germany). The main result of the clamp was whole-body insulin-stimulated glucose disposal at steady state (*M*-value) (mg glucose/kg body weight/min).

### Biochemical Measurements

Fasting plasma glucose, plasma lipids, and creatinine were measured with enzymatic-colorimetric assays (Biosystems, Spain). Glycated hemoglobin A1c (HbA1c) was determined using a National Glycohemoglobin Standardization Program-certified boronate affinity technique (NycoCard^®^ Reader II, Alere Technologies, Norway).

Myonectin was measured with the Aviscera Bioscience Myonectin ELISA (Cat# SK00393-10, dynamic range 8–5,000 ng/mL, intra-assay CV: 6.0–8.0%, interassay CV: 12.0–14.0%), myostatin with the R&D systems GDF-8/Myostatin Quantikine ELISA (Cat#DGDF80, dynamic range 31.3–2,000 pg/mL, intra-assay CV: 1.8–5.4%, interassay CV: 3.1–6.0%) and FGF-21 with the R&D Systems Human FGF-21 Quantikine ELISA (Cat #DF2100, dynamic range 31.3–2,000 pg/mL, intra-assay CV: 2.9–3.9%, interassay CV: 5.2–10.9%). hsCRP was measured using R&D Systems Human C-Reactive Protein/CRP Immunoassay (CAT# DCRP00, dynamic range 0.010–50 ng/mL, intra-assay CV: 3.8–8.3%, interassay CV: 6.0–7.0%). All biomarker immunoassays were based on double-sandwich or competitive-ELISA techniques. Absorbance determinations were done in a BioTek Synergy HT Reader^®^ using the appropriate wavelength according to protocol specifications. All measurements were performed in duplicate. Procedures were executed at the Diabetes, Lipids and Metabolism laboratory of Universidad de Los Andes, following current institutional biosafety protocols.

### Statistical Analyses

We log-transformed plasma triglycerides before statistical analyses and estimated glomerular filtration rate using the Modified Diet for Renal Disease equation (20). Using plasma glucose and insulin values from the 5-point OGTT, we calculated IR indices based on fasting values: HOMA-IR (higher values indicate more IR) (21), quantitative insulin sensitivity check index (QUICKI, higher values indicate less IR) (22); IR indices based on post-glucose challenge values: Incremental area under the insulin curve (iAUCins, higher values numbers indicate more IR), ISI-Gutt (higher values numbers indicate less IR) (23, 24). These are the mathematical expressions of the IR indices:

– HOMA-IR:
  HOMA-IR:[FPI(microU/mL)×FPG(mmol/L)]/22.5.
– Insulin sensitivity index by Gutt (ISI):
  $$\begin{array}{l} \text{ISI}:(\{75,000+\text{[FPG}(\text{mg/L})-120\text{ min glucose }(\text{mg/L})]\}\times 0.19\times \text{body weight (kg))/}120\text{ min}/\\ \;[\text{Mean plasma glucose}(\text{mg/L})]/\text{[log mean serum insulin }(\text{microU/mL})]. \end{array}$$
– Corrected insulin response at 30 min (CIR-30):
  $$\begin{array}{l} \text{CIR-30:}(100\times 30\text{ min insulin})/\{30\text{ min glucose }(\text{mg/dL})\times [30\text{ min glucose }(\text{mg/dL)}-70(\text{mg/dL})]\}. \end{array}$$
– QUICKI:
  QUICKI: 1/(Log fasting glucose [mmol/L]+Log fasting insulin [μU/mL]).
– iAUCins, calculated by the trapezoid method (23).

Comparisons of numerical variables between groups were made with Student’s *t*-tests, after verification of normality and homosedasticity. Comparisons of categorical variables between groups were made with chi-square tests. Participants were placed in quartiles of IR indices and the mean concentration of each myokine was calculated for each quartile. Reported *p-*values for trend correspond to those associated with the coefficient of the slope in a linear regression in which mean values in each of the IR index quartiles were the independent variables, and mean concentrations of each myokine in each quartile were the dependent variable. The correlations between IR indices and plasma myokines, and between clinical variables and plasma myokines levels were examined using Spearman’s correlation coefficients. All statistical tests were two-tailed at a significance level of 0.05. Analyses were performed with Statistical Package for Social Sciences version 23.0 (SPSS Inc., Chicago, IL, USA).

## Results

The study sample comprised 81 participants (57% female). Mean age was 51.4 years, BMI 27.6 kg/m^2^ for men and 25.8 kg/m^2^ for women, abdominal circumference 95.1 cm for men and 82.5 cm for women. Mean systolic blood pressure (SBP), total, LDL, and HDL cholesterol, creatinine, and estimated glomerular filtration rate were within normal ranges (Table 1). Plasma myonectin did not differ significantly by sex. Plasma myostatin and FGF-21 were significantly higher in men (*p* = 0.017 for myostatin, *p* = 0.044 for FGF-21). However, these differences might only reflect a different muscle mass in men versus women. When plasma concentrations of myokines were adjusted by LBM (ng/mL of myokine per kg of LBM), only myonectin concentrations were different between sexes: women had significantly higher myonectin per kg of LBM than men (4.37 ng/mL/kg LBM versus 3.15 ng/mL/kg LBM, *p* = 0.004). Among the OGTT-derived indices, ISI-Gutt exhibited the highest correlation with clamp-derived glucose disposal (*r* = 0.52, *p* = 0.023). ISI-Gutt was also the only OGTT-derived significantly correlated with all other IR indices.

**Table 1:** Characteristics of study participants.

	Total (*n* = 81)
Age (years)	51.4 ± 10.1
Weight (kg)	70.4 ± 13.8
Height (cm)	161.9 ± 8.9
Body mass index (kg/m^2^)	26.7 ± 4.1
Body water percent (%)	49.8 ± 4.8
Body fat percent (%)	31.1 ± 7.3
Visceral fat percent (%)	8.9 ± 3.8
Lean mass percent (%)	65.9 ± 7.2
Bone mass (kg)	2.4 ± 0.5
Abdominal circumference (cm)	88.2 ± 12.7
Systolic blood pressure (mmHg)	118 ± 15.4
Diastolic blood pressure (mmHg)	71.4 ± 17.3
Fasting glucose (mmol/L)	5.32 ± 0.54
Glycated hemoglobin (HbA1c) (%)	5.5 ± 1.4
Creatinine (μmol/L)	71.6 ± 15.0
Total cholesterol (mmol/L)	5.17 ± 1.27
Triglycerides (mmol/L)	1.76 ± 0.88
HDL cholesterol (mmol/L)	1.14 ± 0.32
LDL cholesterol (mmol/L)	3.26 ± 1.18
C-reactive protein (mmol/L)	24.2 ± 9.5
HOMA-IR	4.51 ± 4.41
log(HOMA-IR)	0.51 ± 0.33
HOMA-B	226.9 ± 253.4
iAUCins (μUI min/mL)	4,852 ± 5,850
QUICKI	0.324 ± 0.035
M value (mg/kg body weight/min)	5.20 ± 1.88
Myonectin (ng/mL)	170.0 ± 67.8
FGF-21 (pg/mL)	185.3 ± 128.4
Myostatin (pg/mL)	2,963 ± 1,878

*Data are mean ± SD unless indicated otherwise. M-value: whole-body steady-state glucose disposal in the hyperinsulinemic-euglycemic clamp*.

*HOMA-IR, homeostasis model assessment of insulin resistance; HOMA-B, homeostasis model assessment-beta cell; QUICKI, quantitative insulin sensitivity check index; iAUCins, incremental area under the insulin curve; FGF-21, fibroblast grow factor*.

### Correlation between Myokines and Clinical Variables

Plasma myokines were significantly correlated with each other (for myonectin and FGF-21 *r* = 0.35, *p* = 0.003; for myonectin and myostatin *p* = 0.38, *p* = 0.001; for FGF-21 and myostatin *r* = 0.36, *p* = 0.001). Myonectin did not correlate significantly with any clinical IR feature. Myostatin showed a negative correlation with body fat percent (*r* = −0.36, p = 0.001), and a positive correlation with LBM percent (*r* = 0.35, *p* = 0.001) (Table 2). Similarly, FGF-21 showed a positive correlation with LBM percent (*r* = 0.37, *p* = 0.001).

**Table 2:** Spearman’s correlation coefficients between plasma myokines and clinical surrogates of insulin resistance.

	FGF-21		Myonectin		Myostatin	

	*r*	*p*	*r*	*p*	*r*	*p*
BMI	−0.03	0.78	−0.10	0.42	−0.028	0.80
Body fat percent	−0.32	0.005	−0.08	0.52	−0.36	0.001^a^
Abdominal fat percent	−0.06	0.59	0.01	0.92	0.120	0.29
Lean body mass percent	0.37	0.001^a^	0.14	0.22	0.35	0.001^a^
Waist circumference	0.07	0.55	−0.10	0.42	0.013	0.91
Systolic blood pressure	0.00	0.98	−0.08	0.52	−0.104	0.36
Fasting plasma glucose	−0.01	0.93	−0.11	0.34	−0.064	0.58
HbA1c	0.19	0.13	0.15	0.25	0.35	0.004
Log (triglycerides)	0.00	0.99	−0.05	0.68	0.030	0.79
HDL cholesterol	−0.05	0.69	−0.03	0.81	−0.076	0.50
LDL cholesterol	−0.05	0.65	−0.03	0.82	0.030	0.79
hsCRP	−0.13	0.25	0.01	0.92	−0.095	0.40
Fasting insulinemia	−0.13	0.28	−0.01	0.92	0.142	0.21

*^a^Significant at a Bonferroni-adjusted significance level of 0.00384*.

*BMI, body mass index; hsCRP, high sensitivity C-reactive protein*.

### Plasma Myokines and IR/Insulin Secretion

Characteristics of the random subsample of patients who underwent the hyperinsulinemic–euglycemic clamp did not differ significantly from those of the complete study sample (Table 3).

**Table 3:** Comparison of demographic, clinical, and laboratory characteristics of study participants who underwent or did not undergo the hyperinsulinemic–euglycemic clamp.

	Clamp (*n* = 21)	No clamp (*n* = 60)	*p*
Age	52.6 ± 7.48	51.61 ± 11.12	0.654
Weight	69.97 ± 10.38	70.06 ± 15.21	0.977
Height	162.74 ± 9.35	161.66 ± 8.75	0.652
BMI	26.32 ± 2.3	26.69 ± 4.76	0.648
% body water	49.75 ± 4.39	49.8 ± 4.98	0.964
% body fat	31.37 ± 7.04	30.98 ± 7.34	0.836
% abdominal fat	8.62 ± 2.89	8.95 ± 4.13	0.694
Lean mass (kg)	46.31 ± 9.09	45.78 ± 9.74	0.823
Lean mass (%)	66.1 ± 6.45	65.82 ± 7.43	0.908
Bone mass	2.44 ± 0.46	2.82 ± 3.08	0.358
Abdominal girth	86.21 ± 8.12	88.86 ± 13.89	0.326
Systolic BP	115.9 ± 11.48	118.68 ± 16.52	0.418
Diastolic BP	74.65 ± 9.75	74.47 ± 11.1	0.948
Fasting capillary glucose (mmol/L)	5.30 ± 0.39	5.34 ± 0.59	0.772
HbA1c (%)	5.37 ± 0.69	5.73 ± 1.49	0.180
Creatinine (μmol/L)	73.5 ± 14.2	71.7 ± 15.0	0.621
Total cholesterol (mmol/L)	5.03 ± 1.33	5.18 ± 1.23	0.664
Log(triglycerides)	2.17 ± 1.87	2.20 ± 1.89	0.568
HDLc (mmol/L)	1.06 ± 0.27	1.16 ± 0.33	0.180
LDLc (mmol/L)	3.24 ± 1.19	3.23 ± 1.16	0.972
C-reactive protein (mmol/L)	25.1 ± 13.5	31.1 ± 45.1	0.462
Fasting glucose (mmol/L)	5.30 ± 0.38	5.36 ± 0.58	0.634
Glucose at 30 min (mmol/L)	7.48 ± 1.20	7.45 ± 2.1	0.953
Glucose at 60 min (mmol/L)	7.53 ± 1.69	7.86 ± 2.73	0.517
Glucose at 90 min (mmol/L)	6.46 ± 1.54	7.20 ± 2.63	0.130
Glucose at 120 min (mmol/L)	6.14 ± 1.10	6.59 ± 2.57	0.280
Basal insulinemia (pmol/L)	167.5 ± 166.1	118.7 ± 107.2	0.229
Insulinemia at 30 min (pmol/L)	472.5 ± 451.1	503.3 ± 749.2	0.827
Insulinemia at 60 min (pmol/L)	564.9 ± 431.7	502.7 ± 509.9	0.598
Insulinemia at 90 min (pmol/L)	419.9 ± 341.4	341.2 ± 365.8	0.388
HOMA-IR	5.62 ± 5.56	4.1 ± 3.87	0.270
Log (Homa)	0.59 ± 0.35	0.49 ± 0.33	0.263
HOMA-B	310.31 ± 397.49	196.7 ± 165.28	0.227
ISI (Gutt)	70.61 ± 17.48	74 ± 22.49	0.492
QUICKI	0.32 ± 0.03	0.33 ± 0.03	0.240
iAUC-insulin	5,207 ± 4,840	4,724 ± 6,224	0.723

*HOMA-IR, homeostasis model assessment of insulin resistance; HOMA-B, homeostasis model assessment-beta cell; QUICKI, quantitative insulin sensitivity check index; iAUCins, incremental area under the insulin curve; ISI-Gutt, insulin sensitivity index by Gutt; FGF-21, fibroblast grow factor; BMI, body mass index*.

**Figure 1:**
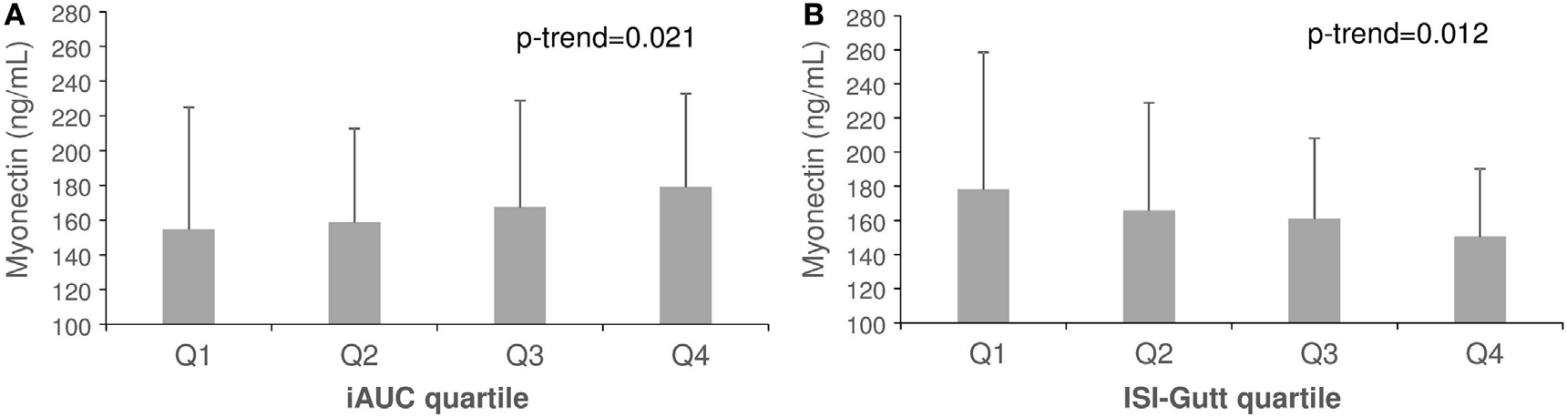
Mean concentrations of myonectin across quartiles of incremental area under the insulin curve **(A)** and insulin sensitivity index **(B)**. Error bars represent SDs.

**Figure 2:**
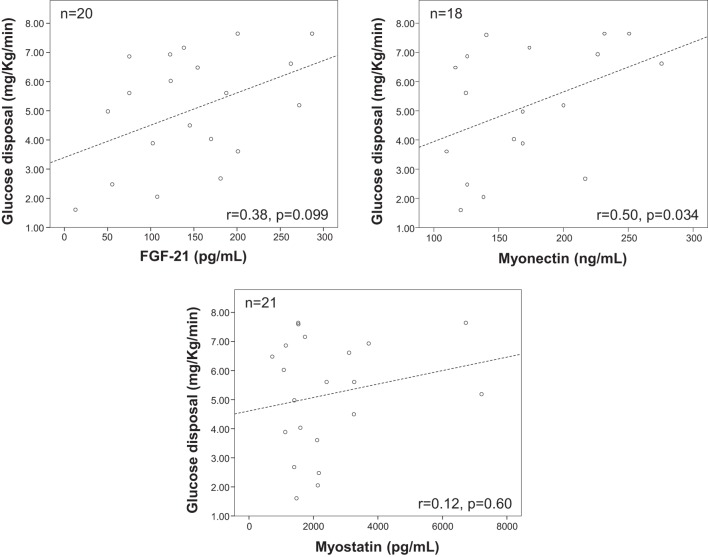
Scatterplots of plasma myonectin concentrations versus whole body insulin-stimulated glucose disposal at steady state in the hyperinsulinemic-euglycemic clamp. *r*-Values are Spearman’s correlation coefficients and their associated *p*-values.

Plasma myonectin was positively associated with IR. The univariate Spearman linear correlation coefficient between myonectin and ISI was *r* = −0.12 (*p* = 0.29), but between myonectin and iAUCins it was *r* = 0.24 (*p* = 0.04). Myonectin levels increased linearly across quartiles of the iAUCins (Q1: 155 ng/mL, Q4: 179 ng/mL, *p*-trend = 0.021), and decreased linearly across quartiles of the ISI (Q1: 178 ng/mL, Q4: 150 ng/mL, *p*-trend = 0.012) (Figure 1). Unexpectedly, myonectin also showed a univariate positive correlation with M value in the hyperinsulinemic-euglycemic clamp (*r* = 0.50, *p* = 0.034) (Table 4; Figure 2). However, after adjustment for key determinants of IR (age, sex, and BMI) in multiple linear regression models, only the negative association of myonectin with the ISI persisted [normalized coefficient (nBeta) = −0.235, *p* = 0.023], while the associations with iAUCins and insulin-stimulated glucose uptake disappeared (*p* = 0.30 and *p* = 0.11, respectively). When percent body fat replaced BMI as a measure of body adiposity in multivariate models, the negative association of myonectin with the ISI was not modified (nBeta = −0.240, *p* = 0.024).

**Table 4:** Mean values of plasma myokines across quartiles of insulin resistance and insulin secretion indices from the OGTT.

	HOMA-IR			iAUCins			ISI-Gutt			QUICKI		
	Q1	Q4	*p*-Trend	Q1	Q4	*p*-Trend	Q1	Q4	*p*-Trend	Q1	Q4	*p*-Trend
Myonectin (ng/mL)	159 (48)	180.6 (80)	0.16	155 (70)	179 (54)	0.021	179 (81)	150 (40)	0.012	179 (82)	159 (48)	0.15
Myostatin (pg/mL)	2,687 (1,770)	3,631 (2,154)	0.49	2,891 (1,702)	3,391 (2,145)	0.42	3,179 (1,894)	2,964 (1,684)	0.53	3,415 (1,976)	2,688 (1,770)	0.57
FGF-21 (pg/mL)	170 (94)	163.8 (103)	0.82	(169) (90)	241 (193)	0.16	180 (109)	165 (93)	0.62	166 (106)	170 (94)	0.85

*Data are mean (SD)*.

*HOMA-IR, homeostasis model assessment of insulin resistance; QUICKI, quantitative insulin sensitivity check index; iAUCins, incremental area under the insulin curve; ISI-Gutt, insulin sensitivity index by Gutt*.

The univariate Spearman’s correlation coefficient between myostatin and ISI was *r* = −0.06 (*p* = 0.61), and between myonectin and iAUCins it was *r* = 0.13 (*p* = 0.25). Myostatin did not change systematically across quartiles of any of the indices reflecting IR, but trended toward a positive association with the iAUCins in the multivariate-adjusted model (standardized beta = −0.206, *p* = 0.072).

Fibroblast-derived growth factor 21 changed across quartiles of the iAUCins (Q1 = 169.3, Q4 = 240.6), but this trend did not reach statistical significance (*p*-trend = 0.16). Similar to myonectin, FGF-21 had a positive albeit non-significant correlation with glucose disposal in the clamp (*r* = 0.38, *p* = 0.099) (Table 3), that was also evident in the adjusted linear model (standardized beta = 0.476, *p* = 0.091). The univariate Spearman’s correlation coefficient between FGF-21 and ISI was *r* = −0.02 (*p* = 0.87), while between myonectin and iAUCins it was *r* = 0.16 (*p* = 0.18).

## Discussion

In this study, we report the association between plasma concentrations of various myokines and objectively measured IR in humans. We found that despite their mutual correlation, myokines exhibit different associations with IR.

In our sample of subjects without known diabetes, myonectin concentrations were positively associated with measures of IR like the iAUCins and negatively associated with insulin sensitivity measures like the ISI-Gutt. Adjustment for major covariates rendered the association with iAUCins non-significant, but the negative association with ISI persisted. In mice, myonectin synthesis and secretion is induced by the hormonal response to the postabsorptive state. Myonectin is able to induce fatty acid uptake by liver and adipose tissue (8). A previous study in 28 women who performed aerobic exercise for 10 weeks identified a positive correlation between semi-quantitatively determined plasma myonectin (western blot) and HOMA-IR (9). Integration of our findings with this prior evidence suggests that myonectin may constitute a muscle-based nutrient-sensing mechanism that works to increase nutrient uptake in other tissues. Thus, increased myonectin levels in the presence of IR may represent an attempt at a compensatory mechanism.

Recombinant myonectin has shown to reduce the concentrations of FFA in mice (8), so it is conceivable that myonectin-induced FFA uptake would reduce the deleterious effect of FFA on systemic insulin sensitivity. We did not measure FFA in our study participants, so we do not have enough information to either confirm or rule out this hypothesis, which would reinforce a role for myonectin as a compensatory mechanism against IR. Future studies of the impact of myonectin on IR in humans may benefit from simultaneous measurement of FFA.

Yet another possibility is that high body adiposity is a common precursor to both IR and increased myonectin. Obesity is characterized by hyperleptinemia, and leptin administration has demonstrated to increase transcription of the myonectin mRNA in mouse myocytes (25). However, the fact that the association between plasma myonectin and ISI was independent of BMI or percent body fat in multivariate models suggests that the link between myonectin and IR is at least partially independent of the leptin axis.

Myostatin on the other hand was not significantly correlated with IR. In a study of 10 obese individuals (26), a 6-month aerobic exercise intervention induced a reduction in both muscle and plasma myostatin and an improvement in IR measured by the intravenous glucose tolerance test-minimal model. In the same report, myostatin administration to mice directly induced IR (27). A study in humans with or without diabetes found an association between myostatin and the HOMA-IR, though only among participants without diabetes (27), and only at the gene expression level, not for the circulating protein. Contrastingly, plasma myostatin did not correlate with one-year weight loss among obese participants of a non-surgical weight loss program (28). Also against a direct involvement of myostatin in human IR, a study of 246 subjects found people with more severe metabolic syndrome to have lower, not higher plasma myostatin (7). Thus, evidence about the involvement of myostatin on human IR is conflicting. Compared to prior reports in younger individuals (mean age 20 years), our study subjects exhibited lower average plasma myostatin concentrations (2.96 ng/mL in our study versus 12.3 ng/mL) (29). In fact, plasma myostatin decreases with age (29), as it happens with muscle mass. A host of findings suggest that even though myostatin secretion is proportional to total muscle mass, its effect may constitute a negative-feedback signal that reduces differentiation of myotubes (3, 6). The apparent inconsistencies regarding the influence of myostatin on IR may be explained if myostatin action is mostly paracrine and thus not reflected by circulating concentrations.

Fibroblast-derived growth factor 21 levels correlated positively with percent LBM, in accordance with its action as a promoter of thermogenesis and “browning” of adipose tissue in animal models (30). Plasma FGF-21 exhibited a monotonic, yet not linearly significant association with the iAUCins. Despite this pattern, FGF-21 tended toward a positive association with glucose disposal in the clamp, suggesting a failed or overwhelmed compensatory mechanism. Prior evidence points in the same direction, as plasma FGF-21 correlates positively with adiposity, fasting insulin, and triglycerides in humans (14). Patients with diabetes have elevated FGF-21 levels (31), and plasma FGF-21 has been associated with IR measures including the AUCins in community-dwelling individuals (32). Another potential explanation for the increased FGF-21 in obesity and IR is the existence of a FGF-21-resistant state, a hypothesis that has been proven in a diet-induced obesity mouse model (33).

It is interesting that some, but not all myokines displayed a significant association with IR. Despite sharing a common tissue source, myokines possess very different structural and physiological properties. Thus, myostatin induces in muscle cells a set of effects opposite to those of myonectin (3, 6, 8), and a similar pattern maybe expected at the whole-body level. In the case of FGF-21, high plasma levels and resistance to its effects have been documented in rodent models of obesity and diabetes (11, 33). It is conceivable that the secretion of FGF-21 as a compensatory hormone that increases energy expenditure (34), may occur only in the face of massive IR, while in individuals with a more moderate degree of IR it only manifests as the non-significant trend that we detected.

The main limitations of our study include its relatively small number of participants and the cross-sectional nature of the measurement of myokines and IR in a particular group of participants of Hispanic ethnicity, all of which limit the generalizability of our results. Another important limitation is that our study design does not allow us to infer the mechanism(s) explaining the observed association between myonectin and IR. However, our study is one small step closer to interventional studies in humans that may evaluate the direct influence of myokines on insulin action and metabolic disease.

In summary, we simultaneously studied three myokines in a sample of adult individuals without known diabetes and comprising a wide range of age and body adiposity. Our findings suggest that myonectin and probably FGF-21 constitute an attempt at a compensatory mechanism against IR. The association of myostatin with human IR is less clear and will require further study.

## Ethics Statement

Full name of the ethics committee that approved the study: Comité de Ética Universidad de los Andes, minute 307 of 2014. Consent procedure used for human participants or for animal owners. All study subjects underwent an informed consent procedure and provided written informed consent. Any additional considerations of the study in cases where vulnerable populations were involved, for example minors, persons with disabilities or endangered animal species. Not applicable. The study complied with scientific, technical and administrative norms for health research dictated by resolution 008430—1993 of the Colombian Ministry of Health and with the principles stated by the Declaration of Helsinki.

## Author Contributions

FT contributed to study conception, execution, data collection, statistical analysis, and manuscript writing. JM-R contributed to study conception, execution, data collection, statistical analysis, and manuscript revision. MP-M contributed to study execution, data collection, statistical analysis, and manuscript revision. MR-S, MM-A, JP-C, MM, MA-G, and GT-G contributed to study execution, data collection, and manuscript revision. CM directed the study and contributed to study conception, execution, data collection, statistical analysis, and manuscript writing.

## Conflict of Interest Statement

The authors declare that the research was conducted in the absence of any commercial or financial relationships that could be construed as a potential conflict of interest.
